# Phase II trial of oral S-1 combined with gemcitabine in metastatic pancreatic cancer

**DOI:** 10.1038/sj.bjc.6603168

**Published:** 2006-05-23

**Authors:** K Nakamura, T Yamaguchi, T Ishihara, K Sudo, H Kato, H Saisho

**Affiliations:** 1 Department of Medicine and Clinical Oncology, Graduate School of Medicine, Chiba University, 1-8-1 Inohana, Chuo-ku, Chiba 260-8670, Japan; 2 Research Center Hospital for Charged Particle Therapy, National Institute of Radiological Sciences, Chiba, Japan

**Keywords:** S-1, gemcitabine, metastatic pancreatic cancer, phase II study, high response

## Abstract

We conducted a phase II trial of gemcitabine with S-1, oral fluorouracil (5-FU) prodrug tegafur combined with two modulators, 5-chloro-2, 4-dihydroxypyridine and potassium oxonate, to evaluate the activity and toxicity of such a combination in metastatic pancreatic cancer (MPC) patients. Patients who had pathologically proven pancreatic cancer with metastatic lesions were eligible candidates for entry into the study. S-1 was given orally (30 mg m^−2^) b.i.d. for 14 consecutive days and gemcitabine (1000 mg m^−2^) was given on days 8 and 15. The cycle was repeated every 21 days. We enrolled 33 MPC patients. The median number of cycles was eight (range 1–20). Grade 3–4 toxicities were leucopenia (33%), neutropenia (55%), anaemia (9%), thrombocytopenia (15%), anorexia (6%), fever (9%), and interstitial pneumonia (6%). Objective responses were obtained in 16 patients (one complete response and 15 partial responses; response rate, 48%; 95% confidence interval (CI), 33–65). Median survival and 1-year survival rate were 12.5 months (95% CI, 5.9–19.1) and 54% (95% CI, 36–72), respectively. Combination chemotherapy with GEM and S-1 was well tolerated and yielded a significantly high response rate.

Pancreatic cancer is one of the most frequently observed gastrointestinal cancers and its prognosis remains extremely dismal. It is the fifth leading cause of cancer death in Japan, as well as in the US and European countries (Matsuno *et al*, 2004). The 5-year survival rate is still poor, at less than 10%, which is commonly considered to be linked to the high incidence of metastatic disease even on initial diagnosis, as well as the relative chemoresistance of this tumour. Therefore, innovations in systemic chemotherapy are needed to improve the survival of patients with advanced pancreatic cancer (APC) (Glimelius *et al*, 1996; Evans *et al*, 1997).

Over the past few years, gemcitabine has been the most widely used chemotherapeutic agent in APC and was reported to yield significantly better symptom control of APC than 5-FU in a randomised phase III clinical study (Burris *et al*, 1997). However, the activity of gemcitabine in pancreatic cancer remains modest and there is a clear need to improve its efficacy by combining it with other anticancer drugs.

Chemotherapy combinations for the treatment of pancreatic cancer could involve prolonged or continuous infusion of 5-FU, because the combination of gemcitabine and 5-FU is shown to have a marked synergistic cytotoxic effect against pancreatic cancer cells in *in vitro* assay (Bruckner *et al*, 1998). Oral administration of 5-FU is not effective, owing to the inability to achieve plasma concentration of sufficient magnitude. An interesting way to increase the efficacy of 5-FU is through the inhibition of the degrading enzyme, dihydropyrimidine dehydrogenase (DPD).

S-1 is a new oral fluorinated pyrimidine developed by Taiho Pharmaceutical Co Ltd (Tokyo, Japan). The agent contains tegafur (FT), 5-chloro-2,4-dihydroxypyridine (CDHP) and potassium oxonate (Oxo) in a molar ratio of FT : CDHP: Oxo=1 : 0.4 : 1, based on the biochemical modulation of 5-FU (Shirasaka *et al*, 1996a, 1996b). Tegafur, a prodrug of 5-FU, is gradually converted to 5-FU and is rapidly catabolised by DPD in the liver. 5-chloro-2, 4-dihydroxypyridine is a competitive inhibitor of 5-FU catabolism, being about 180 times more potent than uracil in inhibiting DPD (Tatsumi *et al*, 1987). When combined with 5-FU, this results in the prolonged maintenance of 5-FU concentrations, both in plasma and in tumours. In addition, it has been suggested that CDHP has the potential to enhance the antitumour activity of 5-FU against subcutaneous tumours in nude mice, using human pancreas carcinoma cells with a high tumoural DPD activity (Takechi *et al*, 2002). Oxo is an agent that decreases the phosphorylation of 5-FU in the gastrointestinal tract by inhibiting the enzyme pyrimidine phosphoribosyl transferase. Oxo preferentially localises in the gut rather than in the tumour and has a potential biochemical effect on the enzyme pyrimidine phosphoribosyl transferase, thereby selectively inhibiting the formation of 5-FU nucleotides in the gut and theoretically reducing gastrointestinal side effects (Takechi *et al*, 1997).

S-1 has undergone phase I evaluation in Japan, as well as extensive phase II studies in gastric, colon, head and neck and breast cancers, leading to registration in this country for gastric cancer. In phase II studies for advanced gastric cancer conducted in Japan, S-1 showed high response rates of 44–49% (Sakata *et al*, 1998; Koizumi *et al*, 2000). In studies outside of Japan, the phase II studies of S-1 against gastric (Chollet *et al*, 2003) and colorectal cancer (Van den Brande *et al*, 2003) in Europe by the EORTC-Early Clinical Study Group revealed moderate activity. The antitumour activity of S-1 in patients with pancreatic cancer has not yet been investigated outside Japan, but favourable results of S-1 monotherapy have been reported in Japanese early phase II and late phase II studies of patients with APC (Furuse *et al*, 2005; Ueno *et al*, 2005).

The administration of oral S-1 is more convenient and simulates the effect of continuous infusion of 5-FU. The combination of gemcitabine and 5-FU is shown to have a marked synergistic cytotoxic effect against pancreatic cancer cells in *in vitro* assay (Bruckner *et al*, 1998). We anticipated that combination chemotherapy of gemcitabine and S-1 would be effective through the synergistic activity of gemcitabine and 5-FU derived from S-1. Thus, we performed a phase I study to evaluate the safety of treatment combining GEM with S-1 and to determine the MTD of each drug in patients with APC (Nakamura *et al*, 2005). This combination chemotherapy was well tolerated and showed outstanding antitumour activity.

Therefore we conducted a phase II study of this combination chemotherapy in patients with metastatic pancreatic cancer (MPC) and assessed the efficacy and toxicity of this regimen.

## PATIENTS AND METHODS

### End point

The primary end point of this study was to determine the efficacy of a combination of gemcitabine and S-1 in MPC. The secondary end points were to assess toxicity, time to progression, and survival.

### Patient selection

Patients with histopathologically proven APC with distant metastasis were eligible for the study. Other eligibility criteria included: 20–74 years of age, Eastern Cooperative Oncology Group (ECOG) performance status of 2 or less (ambulatory and capable of self-care), estimated life expectancy of more than 2 months, adequate renal function (normal serum creatinine and blood urea nitrogen levels), liver function (total bilirubin level ⩽2.5 times upper normal limit (UNL) or ⩽3 times UNL after biliary drainage if the patient had obstructive jaundice and serum transaminases (GOT, GPT) levels ⩽2.5 times UNL or ⩽3 times UNL), bone marrow reserve (white blood cell count between 4000 and 12 000 mm^−3^, neutrophil count ⩾2000 mm^−3^, platelet count ⩾100 000 mm^−3^ and haemoglobin level ⩾9.5 g dl^−1^) and pulmonary function (*P*aO_2_ ⩾70 mmHg). If the patients had a previous history of cancer treatment, that treatment (tumour resection, chemotherapy, immunotherapy, or radiotherapy) had to have been discontinued for at least 4 weeks before entry into the study. All subjects provided written informed consent.

The exclusion criteria were as follows: pulmonary fibrosis or interstitial pneumonia, marked pericardial effusion, severe heart disease, difficult to control diabetes mellitus, active infection, pregnant or lactating women, women of childbearing age unless using effective contraception, severe drug hypersensitivity, metastases to the central nervous system, severe neurological impairment or mental disorder, active concomitant malignancy, and other serious medical conditions. The patients that have pancreatic cancer with neuroendocrine characteristics were excluded.

This study was approved by the institutional review board of Chiba University Graduate School of Medicine.

### Treatment plan

We gave orally 30 mg m^−2^ S-1 twice daily, after breakfast and dinner for 14 consecutive days (from the evening of day 1 to the morning of day 15) followed by a 1-week break. Each capsule of S-1 contained 20 or 25 mg of FT. Individual doses were rounded down to the nearest pill size less than the calculated dose, given the available formulation. We administered 1000 mg m^−2^ gemcitabine in a 30-min intravenous infusion on days 8 and 15 of each cycle. The cycle was repeated every 21 days.

The dose of S-1 was not adjusted for toxicity, because reducing dose of 30 mg m^−2^ twice daily S-1 could not maintain effective blood concentration as 5-FU and the synergistic activity of gemcitabine and 5-FU derived from S-1 was weakened. Similarly, the dose of infusional 5-FU was fixed, and the dose of gemcitabine was adjusted for toxicity in the report of phase I/II study of gemcitabine combined infusional 5-FU (Hidalgo *et al*, 1999).

Full doses of both drugs were given in cases with grade 0–1 toxicity. If grade 2 toxicity was observed the gemcitabine dose was reduced to 800 mg m^−2^ on days 8 or 15. In cases of grade 3 toxicity, gemcitabine administration was omitted. In cases of grade 4 toxicity, both drugs were stopped and adjourned for 1 week.

When grade 3 toxicity was observed in two consecutive cycles, or when grade 4 toxicity was observed even once, 800 mg m^−2^ gemcitabine and 30 mg m^−2^ twice daily S-1 were administered for subsequent cycles. When grade 3 or 4 toxicity was observed even at those doses, further reduction to 600 mg m^−2^ gemcitabine and 30 mg m^−2^ twice daily S-1 were administered for subsequent cycles. We abandoned this treatment when grade 3 or 4 toxicity was observed at that dose.

### Pretreatment and follow-up studies

Pretreatment evaluation consisted of baseline studies including medical history, physical examination, WHO performance status assessment, blood chemistries, urine analysis, electrocardiograms, CA19-9 serum levels. Chest X-ray and abdominal computed tomography (CT) were performed within the period of 2 weeks before starting chemotherapy in order to accurately define the extent of the disease and the target lesions. Measurable disease was defined as a bidimensionally measurable lesion 10 mm or more in size on spiral CT scan. Patients were re-evaluated every two cycles (i.e. every 6 weeks) and then every 2 months after the withdrawal of the protocol. Blood cell counts were performed weekly during treatment and serum chemistry before every new cycle.

The National Cancer Institute (NCI) Common Toxicity Criteria (CTC) scale (version 2.0) was used to evaluate treatment-related side effects.

### Assessment of efficacy

All patients were included in efficacy measurements on an intent-to-treat basis. Tumour responses were evaluated according to the World Health Organization's criteria (World Health Organization, 1979). A complete response (CR) was defined as the disappearance of all clinical evidence of the tumour for a minimum of 4 weeks. A partial response (PR) was defined as a 50% or greater reduction in the sum of the products of two perpendicular diameters of all measurable lesions for 4 weeks or longer without any evidence of new lesions. Stable disease (SD) was defined as less than a 50% reduction or less than a 25% increase in the sum of the products of the two perpendicular diameters of all lesions for 4 weeks or longer without any evidence of new lesions. Progressive disease (PD) was defined as an increase of 25% or more in the sum of the products of two perpendicular diameters of all lesions, the appearance of any new lesion, or deterioration in clinical status that was consistent with disease progression. To assess objective response, patients were evaluated every two cycles (i.e. every 6 weeks) by three independent radiologists

The time to progression was measured from entry into the trial up to the time when progression or death without evidence of progression was observed.

Overall survival was estimated from the date of first treatment to death or last follow-up visit.

### Statistics

The number of patients required for the study was determined according to the optimal two-stage design. Threshold response rate and expected response rate were 10 and 30%, respectively. The sample size of this trial was 29 patients (*α*- and *β*-error probabilities 0.05 and 0.2, respectively). Time-related parameters were analysed using Kaplan–Meier on an intention-to-treat analysis.

## RESULTS

All 33 patients with APC were registered between September 2003 and February 2005. Of 33 patients, 28 had liver metastasis, six had lung metastasis and one presented with peritoneal carcinomatosis and massive ascites only (Table 1). Although eligibility criteria included patients who had a previous history of cancer treatment (tumour resection, chemotherapy, immunotherapy, or radiotherapy) before entry into the study, in actuality no patients had previously received such treatment.

A total of 278 cycles (median 8, range 1–20) were administered. Eleven patients (33%) received full dose intensity (Table 2).

A total of 22 patients (67%) observed grade 2 or more toxicity needed dose reductions of administration of gemcitabine at least once. However, 13 out of these patients could continue this combination regimen at preplanned dose of 1000 mg m^−2^ of gemcitabine from the subsequent cycles. The other nine (27%) patients still continued at reduced dose of gemcitabine for the subsequent cycles. Thus, 24 (73%) of all 33 patients did not require one or more step of dose reduction of administration of gemcitabine for all cycles.

### Efficacy and survival

Results are shown in Table 3. An overall objective response was observed in 16: one CR and 15 PR, and the overall response rate was thus 48% (95% confidence interval (CI), 33–65%). Although early discontinuation of treatment before the first evaluation was caused by early progression in two patients, all responses were confirmed 1 month later. Progressive disease was observed in eight patients (24%) including the two patients.

**Figure 1:**
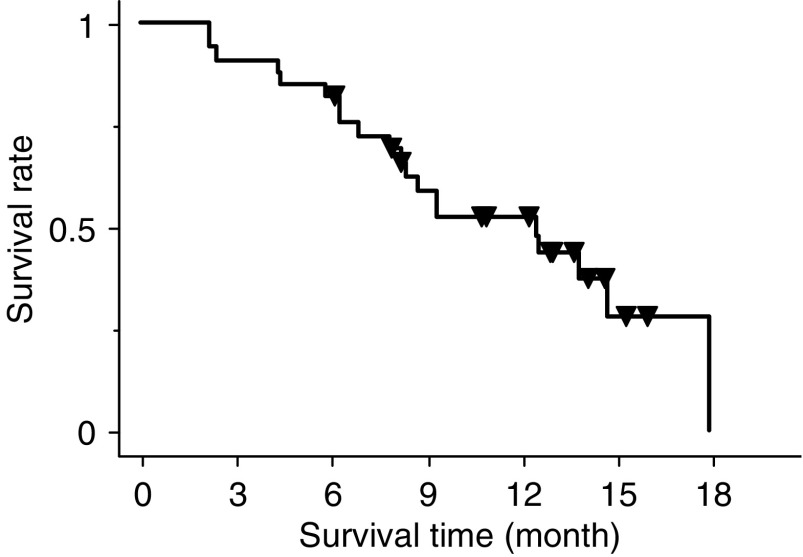
Overall survival curve for all 33 patients. Of 33 patients, 13 are still alive. Median survival time was 12.5 months (95% CI, 5.9–19.1 months). One-year survival rate was 54% (95% CI, 36–72%).

Median time to progression was 5.4 months (95% CI, 2.5–8.4 months). Overall survival was 12.5 months (95% CI, 5.9–19.1 months). The Kaplan–Meier estimate of survival is shown in Figure 1. The 1-year survival rate was 54% (95% CI, 36–72%). Overall, at the time of the last analysis, 20 patients had died, all of them due to progression of disease.

### Toxicity

Maximum toxicity data for the 33 patients during all cycles of this chemotherapy are listed in Table 4. The National Cancer Institute/Common Toxicity Criteria grade 3 or 4 neutropenia, thrombocytopenia and anaemia were observed in 55, 15 and 9% of the patients, respectively, including two cases of febrile neutropenia; relevant grade 3 or 4 nonhaematological toxicities consisted of anorexia, nausea, vomiting and diarrhoea but were very limited. Although a reduction of administration of gemcitabine was needed in two-thirds of the patients in this study because of grade 3 or 4 neutropenia, it was possible to limit grade 3 or 4 neutropenia during all cycles to 18% by reducing the quantity of administration of gemcitabine in subsequent cycles. There was no patient who gave up treatment because of neutropenia. There were two patients who stopped treatment because of interstitial pneumonia. Although grade 1 or 2 rash was observed in 79% of the patients for the first cycle, it had improved by the preventive administration of 4 or 8 mg dexamethasone before administration of gemcitabine for subsequent cycles.

## DISCUSSION

Although the current standard regimen for patients with APC consists of single-agent gemcitabine, the objective responses are low and the median survival benefit is modest in comparison with 5-FU alone. Owing to the activity of gemcitabine, a variety of studies have now assessed its activity in combination with other chemotherapy or novel agents. These studies have shown varying degrees of success, with no combination showing clear evidence of significantly superior activity.

Preliminary favourable results of S-1 in patients with APC have been reported in Japanese early phase II study and late phase II study (Furuse *et al*, 2005; Ueno *et al*, 2005). As yet, the combination regimen of S-1 and gemcitabine for patients with APC has not been investigated. We previously performed a phase I study to evaluate the safety of treatment combining gemcitabine with S-1 to determine the MTD of each drug in patients with APC (Nakamura *et al*, 2005). That study indicated that the recommended dose was 30 mg m^−2^ twice daily of S-1 given orally for 14 consecutive days and 1000 mg m^−2^ gemcitabine given on day 8 and 15, and that the cycle should be repeated every 21 days. The main grade 3–4 toxicities observed during first cycle were neutropenia (33%), anaemia (10%), thrombocytopenia (14%) and anorexia (10%). Responses were one CR (5%) and nine PR (43%) among 21 patients. This combination was well tolerated and showed outstanding antitumuor activity. Therefore, we chose to use this regimen in a phase II study in patients with MPC.

This treatment administration as well as the tolerance profile can be considered as satisfactory regarding the toxicities observed although a reduction of administration of gemcitabine was required in two-thirds of the patients. Myelosuppression, especially neutropenia, frequently seen in the combination of continuous infusion 5-FU and gemcitabine, was predicted as the main toxicity of this study. The incidence of grade 3 or 4 neutropenia was greater than that of other toxicities, but, the incidence of gastrointestinal toxicity during all cycles was low.

Our efficacy results compare well with the other combination regimen in pancreatic cancer although it is difficult to compare phase II studies. Our response rate (48%) was much higher than that observed in gemcitabine combined with infusional 5-FU (19%) (Hidalgo *et al*, 1999) and in gemcitabine combined with capecitabine (19%) (Hess *et al*, 2003) or UFT (16%) (Feliu *et al*, 2000), which is also an oral prodrug of 5-FU, like S-1. Moreover, the median survival (12.5 months) and 1-year survival rate (54%) were favourable notwithstanding all patients in this study having distant metastatic disease.

Moreover, oral administration of S-1, which eliminates the cost and inconvenience of continuous infusion of 5-FU, requiring special pumps and catheters, with their potential risks of infection and thrombosis, also contributes to fewer hospital visits during this outpatient treatment. In fact, 31 of all 33 patients were treated at outpatient clinics. These results indicated that the combination at the recommended doses selected in this study is quite feasible in the outpatient treatment setting.

In conclusion, the results of this study demonstrate the tolerability and effectiveness of gemcitabine combined with oral S-1 in patients with APC. The toxicities observed in this study were mainly haematologic, with mild nonhaematologic toxicity. An encouragingly high response rate was observed. This result is very promising, but the survival benefit in comparison with gemcitabine monotherapy needs to be confirmed in a future randomised clinical trial.

## Figures and Tables

**Table 1:** Patient characteristics

**Median age (range)**	**61 (45–73)**
*Gender*	No. of patients (%)
Male	21 (64)
Female	12 (36)
	
*ECOG PS*	
0	11 (33)
1	20 (61)
2	2 (6)
	
*Stage*	
Locally advanced	0
Metastatic	33 (100)
	
*Prior therapy*	
Tumour resection	0
Radiotherapy	0
Chemotherapy	0
	
*Sites of metastatic disease* a	
Liver	28 (85)
Lung	6 (18)
Peritoneum	1 (3)

a Some were overlapping.

ECOG=Eastern Cooperative Oncology Group.

**Table 2:** Duration of administration and dose intensity of gemcitabine

**No. of patients**	**33**
*No. of cycles*	
Total	278
Median	8
Range	1–20
	
*Relative dose intensity of gemcitabine*	
Average	0.81
Median	0.90
Range	0.43–1.0

**Table 3:** Tumour response

	**Response**				
**No. of patients**	**CR**	**PR**	**SD**	**PD**	**Response rate (%)**
33	1	15	9	8	48 (95% CI: 33–65%)

CR=complete response; PR=partial response; SD=stable disease; PD=progressive disease.

**Table 4:** Maximum toxicity per patient during all cycles

	**Grade 1**		**Grade 2**		**Grade 3**		**Grade 4**	
	**Per patient (%)**	**Per cycle (%)**	**Per patient (%)**	**Per cycle (%)**	**Per patient (%)**	**Per cycle (%)**	**Per patient (%)**	**Per cycle (%)**
Leucopenia	5 (15)	78 (28)	14 (42)	66 (24)	11 (33)	16 (5.8)	0	0
Neutropenia	4 (12)	61 (22)	7 (21)	48 (17)	12 (36)	37 (13)	6 (18)	14 (5.1)
Anaemia	5 (15)	24 (8.7)	13 (39)	38 (14)	3 (9.1)	4 (1.5)	0	0
Thrombocytopenia	10 (30)	38 (14)	13 (39)	22 (8.0)	5 (15)	8 (2.9)	0	0
Anorexia	12 (36)	30 (11)	2 (6.1)	5 (1.8)	2 (6.1)	2 (0.7)	0	0
Nausea	14 (42)	26 (9.5)	2 (6.1)	4 (1.5)	0	0	0	0
Vomiting	4 (12)	6 (2.2)	0	0	0	0	0	0
Diarrhoea	1 (3.0)	2 (0.7)	0	0	0	0	0	0
Rash	14 (42)	15 (5.5)	12 (36)	12 (4.4)	0	0	0	0
Fever	5 (15)	10 (3.6)	0	0	3 (9.1)	3 (1.1)	0	0
Stomatitis	4 (12)	4 (1.5)	1 (3.0)	1 (0.4)	0	0	0	0
Interstitial pneumonia	0	0	0	0	2 (6.1)	2 (0.7)	0	0

The total number of cycles was 278, in a total of 33 patients.
